# Model of the Temperature Influence on Additively Manufactured Carbon Fibre Reinforced Polymer Samples with Embedded Fibre Bragg Grating Sensors

**DOI:** 10.3390/ma15010222

**Published:** 2021-12-28

**Authors:** Torkan Shafighfard, Magdalena Mieloszyk

**Affiliations:** Institute of Fluid Flow Machinery, Polish Academy of Sciences, Fiszera 14, 80-231 Gdansk, Poland; tshafighfard@imp.gda.pl

**Keywords:** Additive Manufacturing, composite, temperature, fused deposition modelling, Finite Element Method, Fibre Bragg Grating

## Abstract

This study investigates the thermo-mechanical behaviour of additively manufactured Carbon Fiber Reinforced Polymer (CFRP) with embedded Fibre Bragg Grating (FBG) sensors with respect to their feasibility for utilising them under thermal loading. This was conducted through the Finite Element Method (FEM) inside an ABAQUS environment. Numerical simulation was complemented by several experimental investigations in order to verify the computational results achieved for the specimens exposed to thermal loading. FBG sensors, incorporated into the material by embedding technique, were employed to measure the strains of the samples subjected to elevated temperatures. It was shown that the strains given by numerical simulation were in good agreement with the experimental investigation except for a few errors due to the defects created within the layers during Additive Manufacturing (AM) process. It was concluded that the embedding FBG sensors were capable of identifying thermo-mechanical strain accurately for 3D-printed composite structures. Therefore, the findings of this article could be further developed for other types of material and loading conditions.

## 1. Introduction

Additive Manufacturing (AM), so-called 3D printing, is a layer by-layer fabrication process in which successive layers of the material are deposited above each other taking advantage of a computer aided design to form the final product [1]. Thanks to the recent development in technology and the current trend toward a sustainable environment [2], it has been widely utilised in various industrial applications such as aeronautical, automotive, dental, architectural, and medical sectors for fabrication of various prototypes, particularly with complex geometries since it provides superior design flexibility compared to conventional manufacturing methods [3]. Therefore, it is believed that the AM will be the third industrial revolution, complementing the production line assembly that dominated manufacturing starting in the previous century [4].

Technology development was not limited to the manufacturing processes. Correlating with the fabrication techniques, the desire toward making the materials more efficient, e.g., smart materials have been among the significant goals of science and industry. Therefore, demand for composite materials has notably been increased during the past decade due to their high stiffness-weight ratio [5], as well as corrosion resistance, decorativeness, and thermal stability. On the other hand, the intrinsic sensing capabilities they own make them suitable for Structural Health Monitoring (SHM) in aerospace and mechanical engineering applications [6].

Meanwhile, since composite materials are subjected to different mechanical and/or thermal loading, which decreases the structural performance, it has been essential to develop a reliable and precise real-time monitoring method in order to obtain information on the structural state of material to improve the performance of composite products [7,8,9]. Surface mounted optical fibre sensors have obtained remarkable attention in the field of experimental stress analysis and health monitoring of composite structures [10]. Fibre Bragg grating (FBG) sensor has been preferred while embedding into composite structures to other types of sensors due to their small dimension and low weight, corrosion resistance as well as multiplexing capability and high sensitivity [11]. It is worth highlighting that the effect of FBG sensors embedding on structural durability is very limited. A FBG sensor is a type of distributed Bragg reflector constructed in a short segment of optical fiber that reflects particular wavelengths of light and transmits all others. The operation of FBG is based on the measurement of the changes in a reflective signal, which is the center wavelength of back-reflected light from a Bragg grating, depending on the effective refractive index of the core and the periodicity of the grating [12]. The deficiency of trusted reliable in-situ monitoring for purposes of controlling the building process and final product quality has prevented AM technology from developing [13], thereby embedding FBG sensors within composite structures is necessary for real-time monitoring of temperature alterations and measurements of induced residual strains. It is also a highly innovative and promising technique in order to identify significant process defects while manufacturing samples [14]. Temperature [15] and strain [16] have been mostly measured in Structural Health Monitoring systems through FBG sensors.

Conventional methods could be utilised in order to embed FBG sensors into composite specimens during manufacturing process [17,18]. Additively manufactured pure polymeric specimens could also contain FBG sensors employing e.g., multi-jet printing [19,20] or Fused Deposition Modelling (FDM) [21,22] techniques. It was shown that the structural performance of the pure polymers could be improved significantly via integrating them with short fibre [23,24,25] and/or continuous fibre [26,27,28], while 3D printing with the FDM method.

Notwithstanding the advantages of the FDM process to fabricate composite structures, it is not totally perfect. The quality and strength of the printed sample are negatively affected by the possible defects corresponding to the FDM method [29,30]. Among these defects, thermal gradients are the most important one that causes the flaws in the specimen during the manufacturing process since it encounters sequential melting and rapid cooling cycles of the deposited material. It can lead to detrimental residual stresses, resulting in delamination failure [31,32]. Hence, the temperature history of the manufactured product via the FDM technique should be studied. Temperature variation has a significant role on assessing the quality of bond formation on the interface of neighbour filaments and mechanical properties of the structure [33]. An experimental study was conducted to measure the internal strains while manufacturing the polymeric structures with FBG sensors [34]. The magnitude of the induced residual strains in FDM built composite structures were measured using FBG sensors and the effect of layer thickness was investigated [35]. The behaviour of the coefficient of thermal expansion for carbon and the glass fiber reinforced polymer specimens as well as the effect of the stacking sequence on the residual strain and temperature profiles were studied [36]. AM technique was utilised for different materials including Carbon Fiber Reinforced Polymers (CFRP) and Polylactic Acid (PLA) to insert FBG sensors at the subsurface of the material or in-depth in various locations with a condition of sensors integrity and reliability of measured values [37]. The internal temperature change of different PLA and CFRP specimens were investigated during the AM process through FBG sensors [38]. FBG sensors were utilised to characterise the deformations and residual strains in additively manufactured plates by FDM [39]. Recently, the thermal strain of the FDM-fabricated composite sample due to its exposition on elevated temperature was studied employing FBG sensors. The numerical analyses were conducted to understand the influence of the elevated temperature on AM composite structure with continuous carbon fibre reinforcement. The analyses considered the influence of the fibre reinforcement occurrence and the thickness of the fibre bundles on matrix thermal stability [40]. On the other hand, the main focus of the current paper is on FBG sensors and their influence on the structure as well as their utility for the strain measurement during temperature elevation. The temperature effect on the mechanical behaviour of the 3D printed CFRP specimens was also studied. 3D printed materials with carbon fibre reinforcement were modeled using the Finite Element Analysis software package. Moreover, this paper expanded the investigation in numerical methods in order to analyse the thermomechanical behaviour of the sample model which was exposed to global thermal loading same as which was carried out in the experimental condition. The simulation results were finally validated using experimental work performed at an environmental chamber using FBG sensors embedded into the CFRP material structure.

Mechanical characterisation of CFRP samples has been studied during the FDM process and has not undergone the 3D printed components itself, in several research articles. Also, a few investigations were carried out for the temperature elevation effect on the mechanical behaviour of composite structures specifically to the heat deflection temperature of PLA. Therefore, in this article, CFRP specimens were additively manufactured through FDM process and tested on an environmental chamber to investigate the effect of the temperature elevation on the mechanical behaviour of those samples. The fabricated specimens were also modelled in the Finite Element Analysis software package, ABAQUS in order to simulate the thermo-mechanical behaviour of the manufactured specimens under thermal loading. The resulted simulation was validated using experimental work performed at an environmental chamber using FBG sensors embedded into the CFRP material structure.

## 2. Samples

### 2.1. Manufacturing Method

The chosen AM method for CFRP material was a modified FDM method developed by project partner (Kaunas University of Technology, Kaunas, Lithuania), who designed their own solution of the printing head [41]. MeCreator 2 3D printer of Geeetech with the printing parameters represented in Table 1, Ref. [41] was utilised in order to fabricate the samples.

### 2.2. Sample

**Figure 1:**
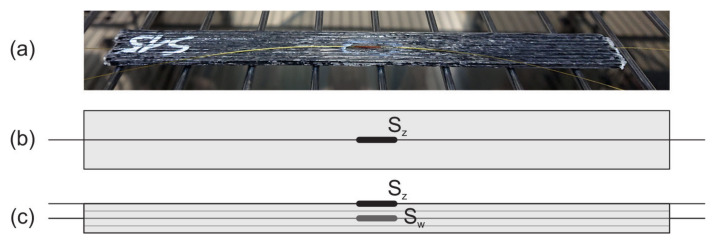
Sample: (**a**) Photograph and scheme, (**b**) top view of the sample, and (**c**) cross-section of the sample; S*_z_*—FBG sensor glued on the surface, S*_w_*—FBG sensor embedded.

The analysed structures are three CFRP specimens with the same dimensions and manufactured using the same AM technique. Their dimensions are listed in Table 2. For the matrix, PLA with DR3D Filament of the diameter 1.75 mm was utilised, while as the reinforcement, the carbon fiber T300B-1000 with the diameter of 1.75 μm from the Toray was chosen. A photograph of one sample and its scheme with marked FBG sensors locations are presented in Figure 1. In the middle of each sample (between the 2nd and the 3rd layer), the FBG sensor (denoted as S*_w_*) was embedded . Additionally, on each sample surface, another sensor (denoted as S*_z_*) was glued using cyanoacrylate glue. Both FBG sensors (10 mm gauge length) were aligned parallel with the carbon fiber direction.

**Figure 2:**
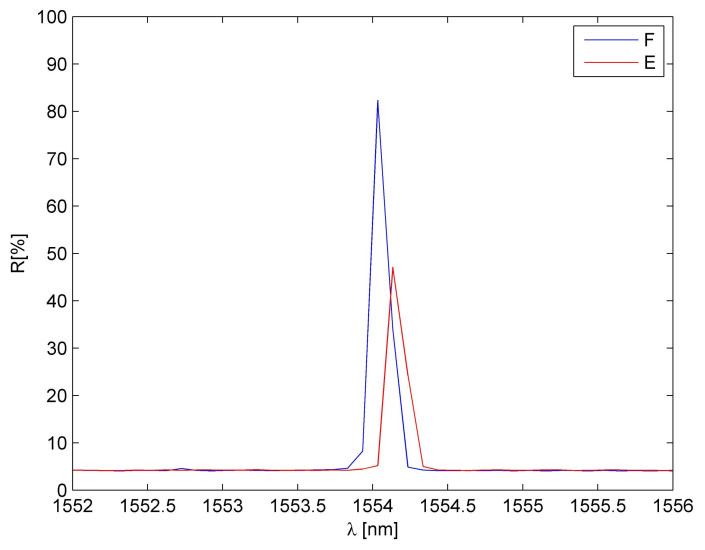
A comparison of FBG sensor spectra: F—free, E—after embedding.

An example of comparison of FBG sensor spectra before and after embedding is presented in Figure 2. It is observed that the embedding process results in the reduction of sensor reflectivity (amplitude reduction) but does not influence the spectrum shape. Therefore, the embedded sensors can be applied for measurements.

The mechanical properties of the materials used for manufacturing the samples are collected in Table 3. The values were given by the manufacturers or achieved from experimental tests [41]. E, ρ, ν, X*_t_*, v, κ, and C are elastic modulus, density, Poisson ratio, failure strength, volume fraction, thermal conductivity, and heat capacity, respectively.

## 3. Experimental and Numerical Investigation

The influence of elevated temperatures on the CFRP samples was analysed experimentally and numerically. The samples were exposed to eight temperature values: 10 °C, 15 °C, 20 °C, 25 °C, 30 °C, 35 °C, 40 °C, and 45 °C. The maximal temperature was related to Tg temperature of the used PLA material. Generally, the Tg of PLA lies between 50 °C and 70 °C [42]. Based on the Dynamic Mechanical Analysis (DMA), performed on the PLA material, it has the relatively low deflection temperature value as of 42 °C [40] .

The investigations were performed under stable Relative Humidity (RH) values, equal to 20%. For both numerical and experimental investigations, the base temperature was equal to 20 °C.

### 3.1. Coefficient of Thermal Expansion of PLA

**Figure 3:**
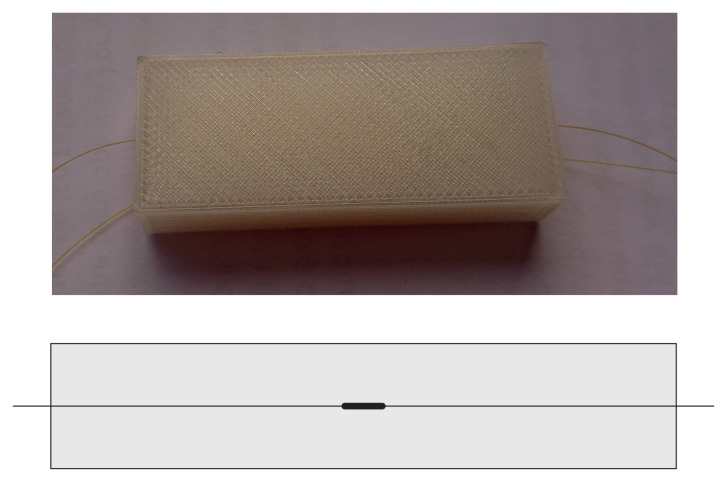
A photograph and cross-section scheme of the PLA sample.

Firstly, the Coefficient of Thermal Expansion (CTE) of the PLA matrix was determined experimentally. It was performed on the sample manufactured under the same manufacturing conditions as the CFRP samples but without adding the fibre reinforcement. The sample dimensions were 50 mm (length) × 20 mm (width) × 10 mm (thickness). In the middle of the sample, an FBG sensor was embedded. The sample photograph and cross-section scheme with marked FBG sensor location is presented in Figure 3.

The measurement was performed in environmental chamber MyDiscovery DM600C (Angelantoni Test Technologies Srl, Massa Martana, Italy). During the investigation, the sample was on the shelf, so it has the possibility to expand in all directions. The measurements were performed using interrogator si425-500 from Micron Optics with a measurement frequency equal to 1 Hz.

Total strain $\varepsilon_{c}$ values for the FBG sensor was calculated using the following equation:

(1) $$\varepsilon_{c}\left(T\right)=\frac{\lambda_{p}\left(T\right)-\lambda_{b}\left(T\right)}{\lambda_{b}\left(T\right)}$$

where $\lambda_{p}$ and $\lambda_{b}$ are measured and base Bragg wavelengths, respectively.

Strain values related to the temperature influence on the PLA material was determined using the following relationship:

(2) $$\varepsilon_{m}\left(T\right)=\varepsilon_{c}\left(T\right)-\varepsilon_{f}\left(T\right)$$

where index *m* is related to the material, while index *f* is linked with the influence of the temperature on the FBG sensor material. Then, for the purpose of determining the relationship between strain and temperature for the PLA material the following equation was applied:

(3) $$\varepsilon_{T}=\frac{1}{n}\sum_{i=1}^{n}\varepsilon_{n}\left(T_{i}\right)\;for\;i=1,\ldots ,8;\;n=n_{1},\ldots ,n_{n};$$

where temperature level $T_{i}$ is related to averaged temperature value from *n* points for stable temperature conditions lasting 300 s. The calculation error for temperature was 0.36 °C, whereas that for the strain was 3.6 × 10^−6^ m/m.

**Figure 4:**
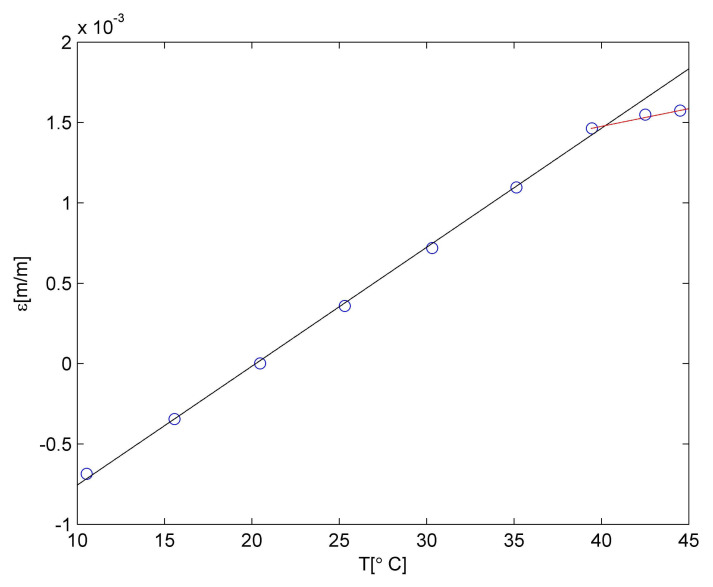
Strain in PLA material due to the temperature influence.

Strain values determined for the PLA material are presented in Figure 4. It is well visible that up to 40 °C, the relationship between strain and temperature is linear. It can be described using the relationship:

(4) $$\varepsilon_{PLA}^{a}\left(T\right)=P_{1}T+P_{2}.$$

The linear approximation is denoted as a black line in Figure 4. The measurement point for 45 °C is not laying on the same straight line, so a correction was introduced. The observed material behaviour is probably related to the deflection temperature of the PLA material. The detailed discussion related to the temperature influence on the PLA material is presented in [40]. So, the relationship between strain and temperature for temperatures higher than 40 °C was approximated using linear relationship but with different polynomial constants. The approximation was denoted as a red line in Figure 4. Both polynomials constants are collected in Table 4.

The thermal expansion coefficient is the first derivative of Equation (4) calculated using the relationship:

(5) $$\alpha \left(T\right)=\frac{\partial \varepsilon (T)}{\partial T}.$$

Therefore, the CTE for the PLA matrix is equal to 7.40 × 10^−5^ m/m °C for temperatures lower than 40 °C and 2.20 × 10^−5^ m/m °C for temperatures higher than 40 °C. Such values were used for numerical calculations.

The observed material behaviour was similar to the one determined by FBG sensor embedded into ABS using the FDM method [22]. There are observed differences between the PLA material behaviour and the previously exterminated M3 crystal, also manufactured using a 3D printer [1]. The relationship between strain and temperature for M3 crystal was described by a second degree polynomial instead of a linear function (also for the temperatures significantly lower than the glass transition temperature). The observed differences between the materials can be not only related to the material properties but also the used manufacturing technique. The M3 crystal samples were manufactured using multi-jet printing (MJP) method, while the PLA was made by FDM. The MJP offers very high accuracy of elements (the layer thickness is equal to 16 μm), but cannot be implemented to manufacturing CFRP elements [1].

### 3.2. Numerical Calculation

**Figure 5:**
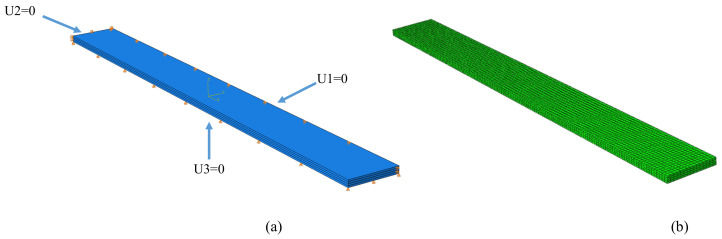
FEM model: (**a**) Boundary conditions and (**b**) meshed model.

The material behaviour corresponding to the effect of the temperature on the CFRP samples was modelled using the Finite Element Method (FEM). Abaqus [43] software was employed in this study for numerical analysis. The ply-by-ply technique was used to model the finite element of the composite laminate. Material orientations with mechanical properties given in Table 2 were assigned to each ply considering their orientation angle for the stated stacking sequence (total of 4 plies). In order to find an optimum number of elements inside the FEM model and avoid high consuming times and achieve an acceptable deviancy, the convergence study was carried out. Therefore, 9000 quadratic hexahedral fully-integrated elements (C3D20RT) with a total of 45,069 nodes were used in the FEM model. The convergence issue was removed while setting seeds size at a similar distance of 1 mm on every edge. It should be mentioned that the bottom part of the sample, which was put on a shelf in the chamber during measurements, was restricted to move in the thickness direction, while one side of the sample was restricted to move in the *y*-direction and another side restricted in the *x*-direction in order to let the sample expanded. The temperature elevation was input to the software as a time-amplitude loading. Finally, the pre-defined temperature of 20 °C was defined for the sample due to the initial temperature of the chamber. The boundary conditions and model were shown in Figure 5.

It was assumed that the temperature corresponding to base conditions was equal to 20 °C. Temperature values were given to the FEM model as an amplitude.

In addition, the emissivity ($\epsilon_{s}=0.96$) was considered for the CFRP sample since it was exposed to thermal radiation that occurred inside an environmental chamber. It should be mentioned that this radiation was applied to all the surfaces since they were in interaction with the ambient. For thermal conductivity, the upper and lower bounds, parallel and serial, were used to estimate the composite properties. The thermal conductivity parameters along the longitudinal and transverse directions to the fiber were provided by:

(6) $$\kappa_{u}=k_{f}v_{f}+k_{m}v_{m}$$

(7) $$\kappa_{t}=\frac{k_{f}k_{m}}{k_{m}v_{f}+k_{f}v_{m}}.$$

In which the $\kappa_{f}$, $\kappa_{m}$, $\nu_{f}$, and $\nu_{m}$ are thermal conductivity of the fiber, thermal conductivity of the matrix, fiber volume fraction, and matrix volume fraction, respectively.

In addition, the coefficient of thermal expansion for longitudinal and transverse direction to the fiber were found respectively by:

(8) $$\alpha_{11}=\frac{v_{f}\alpha_{f}E_{f}+v_{m}\alpha_{m}E_{m}}{v_{f}E_{f}+v_{m}E_{m}}$$

(9) $$\alpha_{22}=\alpha_{f}v_{f}+\alpha_{m}v_{m}.$$

Wherein, $\alpha_{f}$, $\alpha_{m}$, $v_{f}$, and $v_{m}$ are the coefficient of thermal expansion and volume fraction of fiber and matrix, respectively.

### 3.3. Experimental Investigation

Then the experimental investigation of the influence of temperature on the CFRP samples was performed in the environmental chamber. During the investigation, the CFRP samples were kept on the shelf, similarly like the PLA sample. In addition, the same interrogator with the same measurement frequency was applied. The temperature values in the samples location were measured using the FBG temperature probe. The measurements were performed twice and the analyses were performed on the averaged values of the measured Bragg wavelengths.

**Figure 6:**
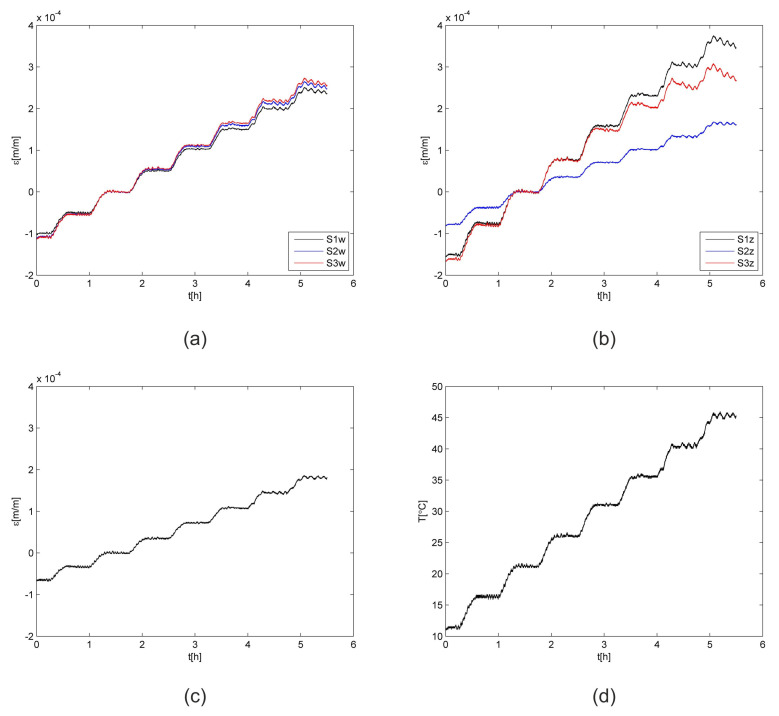
Total strain: (**a**) Embedded sensors, (**b**) sensors on the surfaces, (**c**) free sensor, and (**d**) temperature; S1,S2,S3—CFRP samples.

The total strain values for all FBG sensors are presented in Figure 6. The values were calculated using Equation (1). The average strain variation determined for all sensors was equal to 5 × 10^−6^, while the measurement accuracy of the interrogator was equal to 2 × 10^−6^. It is well visible that all curves registered for strain sensors have the same shape as the temperature curve. All sensors embedded in the samples (S1*_w_*, S2*_w_*, S3*_w_*) have similar strain values, so it can be assumed that they present the averaged strain of the CFRP material. The averaged difference among the strain values determined for the same temperatures is equal to 4 × 10^−6^. While for the sensors mounted on the samples surfaces, differences can be observed. They are related to the FBG sensors location on the carbon fibre and the matrix (S1*_z_* and S3*_z_*) or on the carbon fibre bundle (S2*_z_*). Due to observed differences among sensors, the results were not used for the consecutive calculations and the FEM model evaluation.

**Figure 7:**
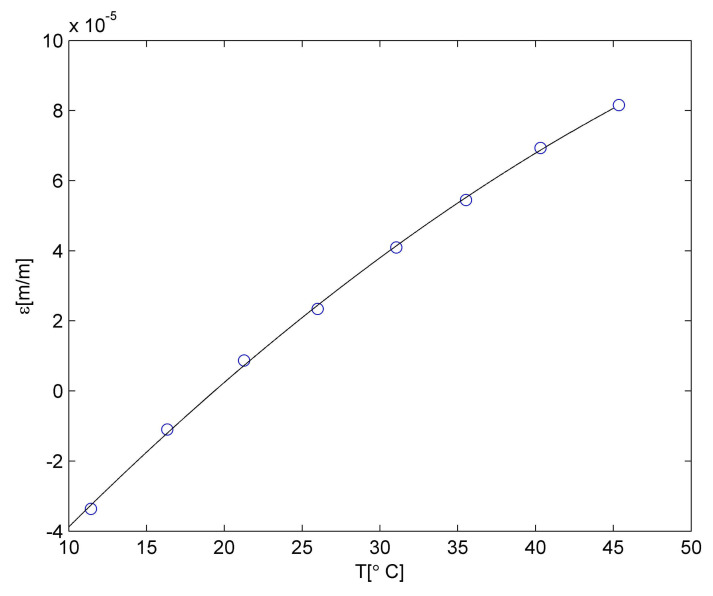
Relationship between strain and temperature for CFRP samples.

Then CFRP strain values were determined using the same procedure as was described for the PLA matrix. The relationship between the temperature and strain is presented in Figure 7.

It is well visible that the relationship is quadratic. For a CFRP 3D-printed sample utilised in this study, the error was calculated to be 2%. This error may be considered statistically negligible and excluded from statistical evaluation.

The average percentage deviation between the experimental and approximation was calculated using Equation (10):

(10) $$\varepsilon_{e}\left(T\right)=\frac{\varepsilon_{E}-\varepsilon_{A}}{\varepsilon_{E}}.$$

In which *E* refers to an experimental and *A* refers to an approximated values of the normal strain, respectively.

**Figure 8:**
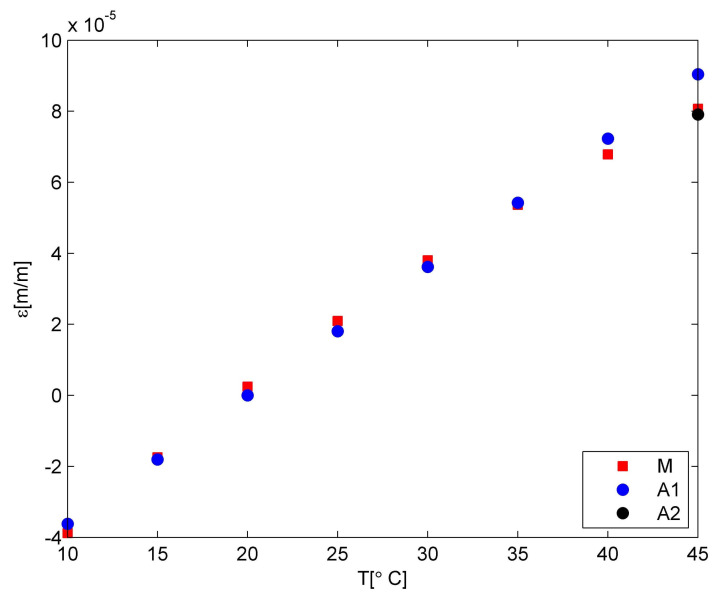
Comparison between model and experiment; M—measurement, A1—model with the first CTE value for PLA, A2—model with the CTE value correction for higher temperatures.

The obtained numerical results of the temperature and strain relationship were compared with the experimental data. The comparative plots are shown in Figure 8. The model values (marked by blue circles) were calculated using the first CTE value of the PLA material. For such an assumption, the average strain difference is equal to 1.20× 10^−6^ for the whole range of temperatures. It is related to 7% differences. Taking into consideration the 45 °C only, the strain difference is equal to 9.74 × 10^−6^. The model correction related to the PLA matrix material characteristic—CTE value of PLA material – allows for decreasing the difference to 1.58 × 10^−6^. Due to the fact that the material behaviour change influenced the one temperature only, the averaged percentage difference was reduced to 5% only.

## 4. Conclusions

A 4-layer composite unidirectional laminate was fabricated employing AM, FDM technique. The numerical results were compared to the averaged strain values for three samples for measurements repeated twice. The effect of the elevated temperature on the thermal strain of the 3D-printed specimens was studied. Both experimental and numerical analyses were carried out at a stable humidity level, that was guaranteed by an environmental chamber utilised for experimental verification.

First of all, the numerical simulation results were compared with the data given by the experimental test. Thermal strains obtained through the ABAQUS software simulation were in good agreement with the corresponding values given by the FBG sensors utilised in this study. The averaged percentage difference was approximately 5% since the material behaviour changes influenced merely the one temperature.

According to the results given by the experimental tests, the strain values for all sensors embedded in the samples (S1*_w_*, S2*_w_*, S3*_w_*) were the same, and the assumption that the averaged strain of the CFRP specimens were presented was justified. The averaged difference among the strain values determined for the same temperatures is equal to 4 × 10^−6^. It was also understood that for the sensors put on the samples surfaces differences were captured which were associated to the FBG sensors location either on the carbon fibre and the matrix (S1*_z_* and S3*_z_*) or on the carbon fibre bundle (S2*_z_*).

On the other hand, it was concluded that the strange behaviour of the material above 40 °C was due to the fact that the PLA material inside the CFRP has not a constant CTE value within the temperature elevation range. It was measured experimentally and the CTE value for 20 °C and 40 °C was constant but it was changed above 40 °C. Therefore, the relationship is linear but the parameter is different and depends upon the ranges.

Therefore, the findings of this article could be considered as motivation for further developing AM techniques of composite structures-embedded FBG sensors. Future work will include the effect of different layer orientations of 3D-printed CFRP samples on the accuracy of the results given by the FBG sensors and thermo-mechanical behaviour of structures.

## Figures and Tables

**Table 1:** Parameters used for fabricating the sample [41].

Speed			Temperature		Extrusion	
First Layer [mm/s]	Printing [mm/s]	Fan [%]	Extruder [°]	Bed [°]	Multiplier [mm]	Width [mm]
1.20	4	50/80/100	200	70	0.6	1.6

**Table 2:** Specimen dimensions.

Sample			Layer		Stacking
Lenght [mm]	Width [mm]	Tickness [mm]	Tickness [mm]	Number	Sequence
150	15	2	0.5	4	[0, 0] s

**Table 3:** Specimen parameters.

	E [GPa]	*ρ* [g/cm^3^]	*ν*	X*_t_* [MPa]	v [%]	*κ* [W/mK]	C [J/kgK]
Fibre	230	176	0.33	3530	18	10.46	794
Matrix	2.315	1.24	0.29	51	82	0.13	1800

**Table 4:** Polynomials constants.

	First Approximation (× 10^−5^)	Second Approximation (× 10^−5^)
*P*_1_	7.40	−149.55
*P*_2_	2.20	59.58
line in Figure 4	black	red

## Data Availability

Data sharing not applicable.
